# Identifying factors associated with of blood pressure using Structural Equation Modeling: evidence from a large Kurdish cohort study in Iran

**DOI:** 10.1186/s12902-022-01244-8

**Published:** 2022-12-30

**Authors:** Farid Najafi, Mehdi Moradinazar, Shahab Rezayan, Reza Azarpazhooh, Parastoo Jamshidi

**Affiliations:** 1 Research Center for Environmental Determinants of Health, School of Public Health, Kermanshah University of Medical Sciences, Kermanshah, Iran; 2 Stroke Prevention and Atherosclerosis Research Center, Robarts Research Institute, Western University, London, ON Canada; 3 School of Medicine, Kermanshah University of Medical Sciences, Kermanshah, Iran

**Keywords:** Blood pressure, Risk factors, Physical activity, Obesity, Diabetes, Structural Equation Modeling

## Abstract

**Background:**

Identifying the risk factors leading to hypertension can help explain why some populations are at a greater risk for developing hypertension than others. The present study seeks to identify the association between the risk factors of hypertension in 35- to 65-year-old participants in western Iran.

**Methods:**

This cross-sectional study was conducted on 9705 adults from baseline data of Ravansar Non-Communicable Disease (RaNCD) cohort study, in the west region of Iran. Each of the latent variables were confirmed by confirmatory factor analysis. Using Structural Equation Modeling (SEM), we assessed the direct and indirect effects of factors associated with blood pressure.

**Results:**

Socioeconomic status (SES), physical activity, mean of serum lipids, obesity, diabetes and family history of hypertension had a diverse impact on the blood pressure, directly and (or) indirectly. The standardized total effect of SES, physical activity, mean of serum lipids, and obesity were -0.09 vs. -0.14, -0.04 vs. -0.04, 0.13 vs. 0.13 and 0.24 vs. 0.15 in men and women, respectively. Diabetes had a direct relationship with the blood pressure in women (0.03).

**Conclusion:**

With regard to control of high blood pressure, public health interventions must target obesity, lifestyle and other risk related to nutritional status such as hyperlipidemia and hyperglycemia in Iranian population and among those with higher SES.

## Introduction

Hypertension is one of the most important risk factors for chronic heart diseases [1, 2]. The incidence of hypertension has increased over the last few decades; the number of adults with hypertension has increased from 595 million in 1975 to 1.13 billion in 2015 [1]. The prevalence of hypertension is predicted to increase 29.2% by 2025 and this increase has largely occurred in low-to middle-income countries [3, 4]. The relationship between high blood pressure and cardiovascular disease and its mortality has been addressed in a number of observational studies [5]. Hypertension shows an independent relationship with the incidence of several cardiovascular events such as stroke, myocardial infarction, heart failure and peripheral arterial disease as well as kidney disease. This relationship is shown for all ages and in all ethnic groups [6, 7].

Hypertension has a wide range of risk factors including, genetic, behavioral and environmental risks [8, 9]. Identifying the risk factors associated with hypertension explains why some populations are at a greater risk for developing hypertension than others. Studies have shown that, after adjustments for age and gender, hypertension are associated with body mass index (BMI), the level of physical activity and genetic factors, smoking, high cholesterol, diabetes mellitus (DM), and other lifestyle factors [10–12]. Nonetheless, these risk factors have not yet been simultaneously examining the intercorrelation of all contributing factors. However, Few studies have been published, addressing the interrelationship between factors associated with mean of blood pressure using structural equation modeling (SEM) [13–18].

By assessing the effect of the latent variables, SEM is the most useful method for the concurrent testing of complex relationships between variables [19].

SEM reduces measurement errors by involving several overt variables for each latent variable. Unlike traditional regression models that treat each covariate in the model as an independent direct effect, SEM allows to test the model with several dependent variables and assess the concurrent direct and indirect effects of several independent variables on the dependent variable.

Available research allows us to hypothesize a model that depicts the relationships between independent factors and blood pressure in terms of direct and indirect (i.e., mediator) effects. The present research which is the first of its kind in Kurdish people, was conducted to use SEM to identify the (direct and indirect) effect of the risk factors associated with of BP in the Ravansar Non-Communicable Disease (RaNCD) cohort study.

## Materials and methods

### Study design and participants

We used data from baseline phase for Ravansar Non Communicable Disease (RaNCD) cohort study, in the west region of Iran. This study started in 2014. RaNCD is a part of Prospective Epidemiological Research Studies in Iran (PERSIAN), conducted in different Iranian ethnicities, in coordination with the Ministry of Health and Medical Education. In fact, 10,000 adults have been recruited for RaNCD cohort study. Ravansar is one of the cities of Kermanshah province. The city of Kermanshah (about 1,000,000 populations), is the center of the province and the largest and most important Kurdish settlement in the western region of Iran. The Ravansar district population is about 50,000 people, mainly from Iranian Kurdish ethnicity. All included participants, have provided oral and written informed consent. Eligibility criteria in the cohort study comprised of being in the age range of 35–65 years, permanent inhabitants of the Ravansar region, and having Iranian nationality [20, 21].

### Inclusion and exclusion criteria

The data used in this study pertained to more than 10,000 participants aged 35 to 65 who had voluntarily entered the study. For the purpose of this study, we excluded those with a clinical history of stroke (50 subjects), myocardial infarction (65 subjects), and renal failure (53 subjects).

### Definitions and measurements

Anthropometric indices including body weight, height, waist to hip ratio and body mass index, waist circumference (WC), were measured according to standard methods. Body weight was measured using Bio-Impedance Analyzer BIA (Inbody770, Inbody Co, Seoul, Korea) with a precision of 0.5 kg. Height was measured by BSM370 (Biospace Co, Seoul, Korea) with the precision of 0.1 cm. BMI was measured by dividing weight (kg) by the square of height (m). The Waist to Hip Ratio (WHR) was calculated by dividing the waist circumference over the hip circumference. WC was measured with a flexible measuring tape at a level midway between the lower rib margin and the iliac crest to the nearest 0.5 cm [22]. The standard physical activity questionnaire of PERSIAN cohort was implemented to assess participants’ physical activity. The questionnaire consisted of 22 questions regarding the amount of an individual’s daily activity. Finally, metabolic equivalent of task (MET), as an indicator for level and measure of physical activity, was extracted and entered into the model. MET is the amount of oxygen consumed at rest (about 3.5 ml 02/kg/min) and is equal to resting metabolic rate. MET for each activity was extracted using a compendium of physical activities [23]. Diabetes was defined as having an FBG $\ge$ 126 mg/dl and/or being on diabetes medication and/or if the diabetes was confirmed by a general practitioner [24]. Self-report family history of hypertension including living and deceased, was any biological blood relatives, ever told by a health professional that they have hypertension. Dyslipidemia was defined by lipoprotein ratios (TC/HDL and LDL/HDL) and added to the model as continuous variables.The outcome variable in this study was a latent variable defined by mean systolic and diastolic BP. BP was measured after 15 min of rest in sitting position. Both arms were measured twice with the cuff size adjusted to the arm circumference. Four BP measurements were taken and the average calculated for both systolic and diastolic blood pressure [25].

### Statistical methods

**Fig. 1:**
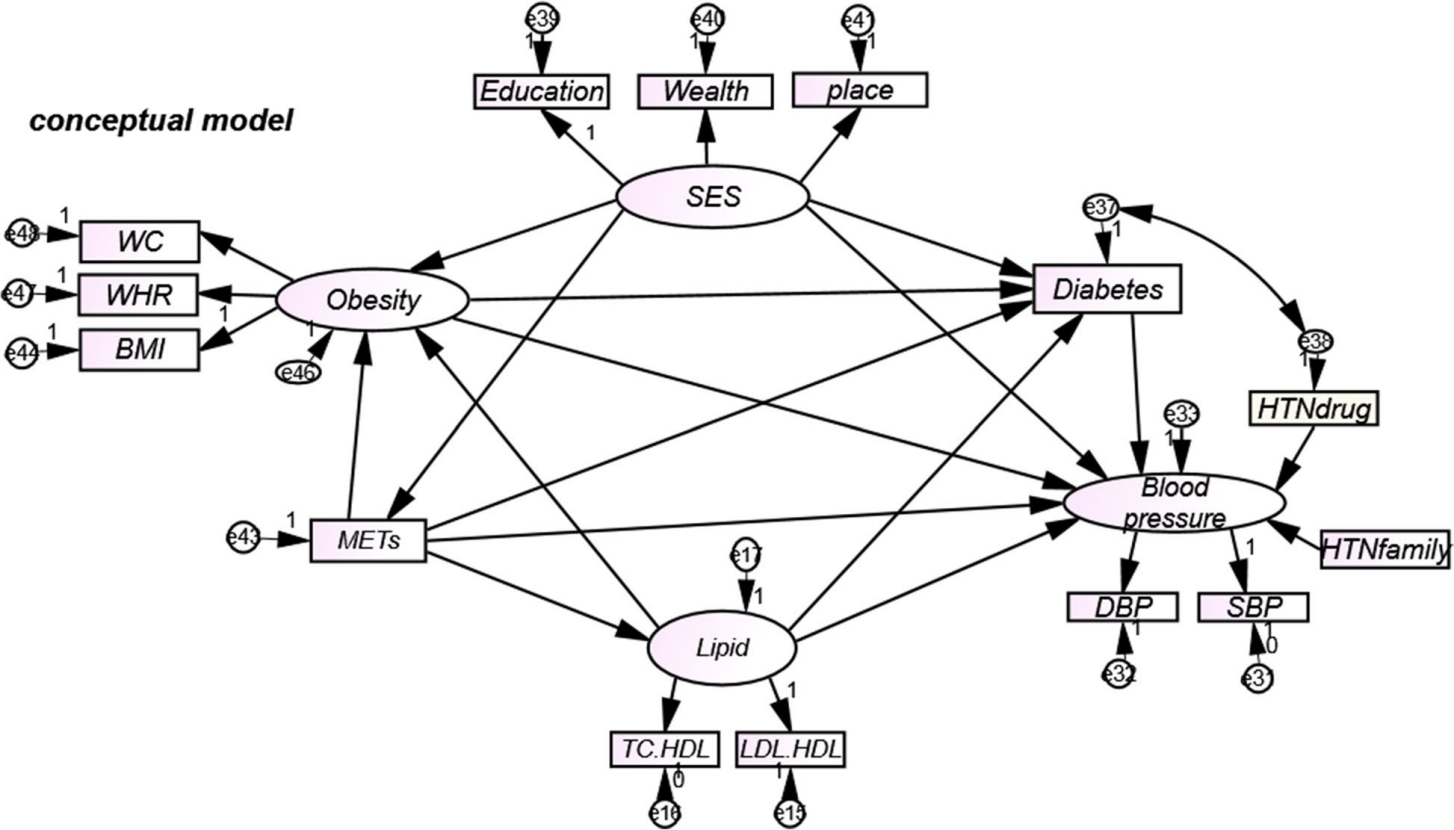
The conceptual model diagram for association between risk factors and blood pressure. SES socioeconomic status, SBP systolic blood pressure, DBP diastolic Blood pressure, WC waist circumference, BMI body mass index, WHR waist to hip ratio, LDL low-density lipoproteins, TC total cholesterol, HDL High-density lipoprotein, HTNfamily Family history of hypertension, HTNdrug antihypertensive drugs

After a comprehensive review of available evidence and consultation with the experts in this field, the conceptual model was developed. Our conceptual model, in fact, represent the hypothesized relationships between different latent and observed variables and their association with our outcome of interest (BP). In SEM, while single-headed arrow represents causal relationship, double-headed arrow represents correlation. Finally, we translate the conceptual model into the statistical model. Accordingly, we first conducted an exploratory factor analysis (EFA) to identify the latent variables underlying the observation variables. The principal component analysis (PCA) and varimax rotation was conducted to estimate the latent variables. The number of the extracted factors was chosen based on the factor eigenvalue ($>$ 1). The economic welfare (wealth) variable was measured using 12 questions regarding housing, car, washing machine, dishwasher, freezer, computer, home appliances and other amenities by PCA method. In order to overcome the problem of having dichotomous variables in PCA, we used polychoric or tetrachoric correlation coefficients and the results of the correlation matrix in PCA [26, 27]. Other variables of SES are education and place of residence (urban or rural areas). Education was categorized to illiterate, first level of education (less than five years of education), second level of education (6–9 years of education) and third level of education (more than 10 years of education). In order to create constructs (or factors), we applied confirmatory factor analysis (CFA) and we constructed an initial SEM. The objective of confirmatory factor analysis is to test whether the data fit a hypothesized measurement model. In the next step, SEM with maximum likelihood estimation (MLE) was applied to assess the conceptual model (Fig. 1). The SEM was used to study the direct and indirect relationships between a set of variables associated with mean BP. In the conceptual model, there are four latent variables including the mean blood pressure, with the indicator of systolic and diastolic blood pressure (SBP, DBP). Considering that BMI, WHR and WC represent obesity indicators, a latent variable named 'obesity ' was constructed. The other latent variable is SES with three indicators: economic prosperity (Wealth); education; and place of residence (place). The fourth latent variable named 'lipid profile' was constructed to reflect TC/HDL and LDL/HDL ratio. Path standardized coefficients (β) as the effect sizes of this model were calculated. For a proper model fit, modifications had to be implemented on the conceptual model over the course of the statistical analysis based on adjustable indices suggested by the software. In our study, due to large sample size, chi-square tests are not suitable for model fitting [28]. However, other fitting indices unrestricted by the sample size are more appropriate. Comparative fit index (FCI), incremental fit index (IFI), and normed fit index (NFI) equal to or greater than 0.90 and Root Mean Square Error of Approximation (RMSEA) equal to less than 0.08 were applied to confirm the fit of the model. Also, data were described using the appropriate method (mean ± standard deviation for quantitative variables and number and percentage (%) for qualitative variable). All of the statistical analysis was performed using AMOS-SPSS 22 and STATA 14.0 (STATA Corp, College Station, TX). *P*-value less than 0.05 was considered as statistically significant.

## Results

### Characteristics of study participants

After excluding those with missing information and people with history of renal failure, stroke and myocardial infarction, 9705 subjects remained for our analyses. The mean age ± SD of the participants was 47.53 ± 8.47 years. About 52% of the participants were female and 47% were male.The mean systolic blood pressure was 107.52 ± 15.45 and the mean diastolic blood pressure was 69.65 ± 9.35. From total, 35% and 16% of women and men had BMI of >  = 30, respectively. In addition, 53% (3257) and 47% (2908) of women and men had waist-to-hip ratio >  = 90, respectively. The general characteristics of the study participants are shown in Table 1.

**Table 1:** Demographic features in 35–65-year-old by sex at RaNCDchort study

	Female	Males	*P* value
Population, n (%)	5108(52.6%)	4597(47.4%)	
Age (years)	47.53 $\pm$ 8.47	46.93 $\pm$ 8.10	< 0.001
Educational level, n(%)			
Illiterate	1788(35.2%)	589(12.8%)	
1–5 years	2395(46.9%)	1320(28.7%)	
6–9 years	505(9.9%)	1108(24.1%)	< 0.001
10 and more	420(8.0%)	1580(34.4%)	
Economic status, n(%)			
poorest	1339(26.2%)	600(13.1%)	
2 ^nd^ poorest	1135(22.2%)	800(17.4%)	< 0.001
middle	1006(19.7%)	933(20.3%)	
2^nd^ richest	909(17.8%)	1040(22.6%)	
Richest	719(14.1%)	1224(26.6%)	
Place			
Rural	2185(42.8%)	1779(38.7%)	< 0.001
Urban	2923(57.2%)	2818(61.3%)	
HTN drug, n(%)			
Yes	755(14.8%)	345(7.5%)	< 0.001
No	4353(85.2%)	4252(92.5%)	
Diabetes, n(%)			
Yes	601(11.8%)	366(8%)	< 0.001
No	4507(88.2%)	4231(92.0%)	
Family history of hypertension			
None	2212(48.1%)	2173(42.5%)	
Second degree	232(5%)	233(4.30%)	< 0.001
First degree	2153(46.8%)	2702(52.9%)	
Blood pressure			
SBP	108.31 $\pm$ 17.01	110.73 $\pm$ 16.07	< 0.001
DBP	69.90 $\pm$ 9.92	71.38 $\pm$ 9.59	
Obesity			< 0.001
BMI(kg/${m}^{2}$)	28.56 $\pm$ 4.86	26.34 $\pm$ 4.06	< 0.001
Waist-to-hip ratio	0.94 $\pm$ 0.05	0.93 $\pm$ 0.04	< 0.001
Waist circumference (cm)	98.30 $\pm$ 11.39	96.16 $\pm$ 9.97	
LDL	102.98 $\pm$ 24.09	101.08 $\pm$ 24.64	
HDL	49.68 $\pm$ 11.42	42.79 $\pm$ 10.07	< 0.001
Total cholesterol	189.0 $\pm$ 39.0	181.64 $\pm$ 36.47	
Lipid profile			
LDL: HDL	0.72 $\pm$ 0.30	0.85 $\pm$ 0.30	
TC: HDL	1.34 $\pm$ 0.25	1.45 $\pm$ 0.26	
MET	39.31 $\pm$ 4.49	42.86 $\pm$ 10.60	

The values outside the parentheses are the number of people and the values inside the parentheses are the percentages. Data are expressed as mean $\pm$ SD. *P* values < 0.05 (student's t test) and (Chi-squared test) compare men with women

*SBP* Systolic blood pressure, *DBP* Diastolic blood pressure, *WC* Waist circumference, *BMI* Body mass index, *WHR* Waist to hip ratio, *LDL* Low-density lipoproteins, *TC* Total cholesterol, *HDL* High-density lipoprotein

### Results of latent analysis variables

Exploratory factor analysis was conducted and the Kaiser-Meyer-Olk statistic was determined to be 0.697, which indicated that the data were suitable for factor analysis. The four latent factors included SES, blood pressure, obesity, lipid profile were extracted with eigenvalue greater than 1. The extraction results of the latent variables are shown in Table 2. In the CFA between the latent variable in the model, correlation and fitting indexes were acceptable: chi-square value (${x}^{2}$) = 624.897, the ratio of ${x}^{2}$ to the degrees of freedom = 25, root mean square error of approximation (RMSEA) = 0.05, comparative fit index (CFI) = 0.98, goodness of fit index (GFI) = 0.987, and tuker- lewis index (TLI) = 0.981 (Table 3).

**Table 2:** Results of latent variables analysis (Varimax rotation)

Latent variable	Indicator variable	Loading coefficient	Cronbach's coefficient
Blood pressure	SBP	0.953	0.863
	DBP	0.958	
SES	Education	0.832	0.589
	Wealth	0.802	
	Place	0.585	
Lipid profile	LDL: HDL	0.973	0.962
	TC: HDL	0.972	
Obesity	BMI	0.916	0.549
	WC	0.905	
	WHR	0.836	

The result of exploratory factor analysis: Kaiser-meyer-olk in measure of sampling adequacy (kmo) = 0.697, Bartlett test of sphericity approx: Chi-square = 56,799.689, *p* $<0.001$

*SES* Socioeconomic status, *SBP* Systolic blood pressure, *DBP* Diastolic Blood pressure, *WC* Waist circumference, *BMI* Body mass index, *WHR* Waist to hip ratio, *LDL* Low-density lipoproteins, *TC* Total cholesterol, *HDL* High-density lipoprotein

**Table 3:** Standardized factor loading of the confirmatory factor analysis

Latent variables	Indicator variables	Loading cofficint
Blood pressure	SBP	0.958
	DBP	0.950
SES	Education	0.644
	Wealth	0.801
	Place	0.335
Lipid profile	LDL: HDL	0.959
	TC: HDL	0.980
Obesity	BMI	0.741
	WC	0.696
	WHR	0.925

*SES* Socioeconomic status, *SBP* Systolic blood pressure, *DBP* Diastolic blood pressure, *WC* Waist circumference, *BMI* Body mass index, *WHR* Waist to hip ratio, *LDL* Low-density lipoproteins, *TC* Total cholesterol, *HDL* High-density lipoprotein

### Results of the model structure

**Fig. 2:**
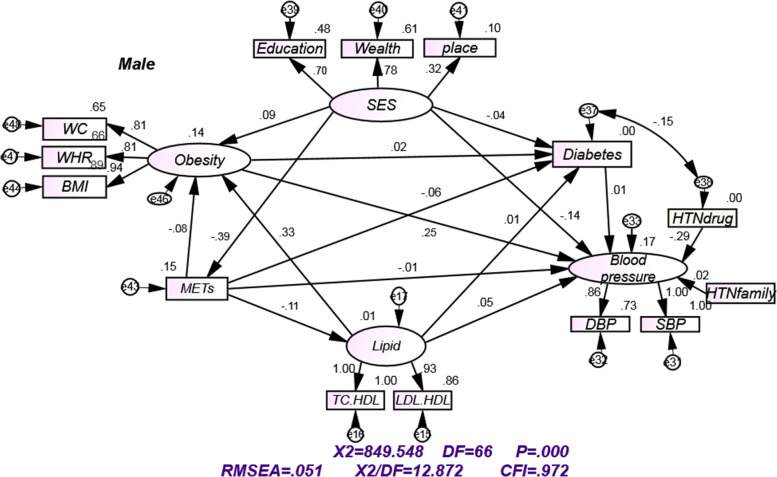
Structural equation models for assessing direct and indirect effects of different risk factors on blood pressure, for males, by standardized path coefficient and goodness of fit indices. "e" represent the errors. SBP systolic blood pressure; DBP diastolic blood pressure; WHR waist to hip ratio; BMI body mass index; SES socioeconomic status, WC waist circumference, LDL low-density lipoproteins, TC total cholesterol, HDL High-density lipoprotein, HTNfamily Family history of hypertension, HTNdrug antihypertensive drugs

**Fig. 3:**
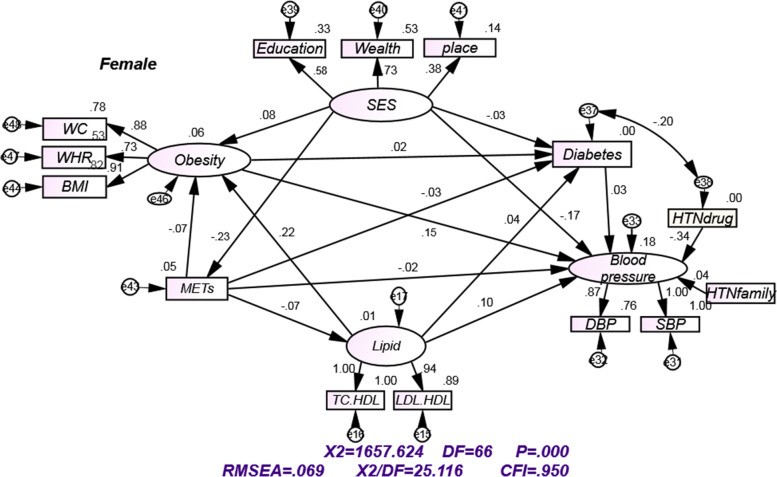
Structural equation models for assessing direct and indirect effects of different risk factors on blood pressure, for females, by standardized path coefficient and goodness of fit indices. "e" represent the errors. SBP systolic blood pressure; DBP diastolic blood pressure; WHR waist to hip ratio; BMI body mass index; SES socioeconomic status, WC waist circumference, LDL low-density lipoproteins, TC total cholesterol, HDL High-density lipoprotein, HTNfamily Family history of hypertension, HTNdrug antihypertensive drugs

The conceptual model of the study included the variables extracted from the results of previous studies and a review of literature plus consultation with experts. The final model was constructed of the different models (Fig. 1) and was determined to be identified as the final model. In figs. 2 and 3, variables of obesity, Diabetes, lipid profile and METs play a mediation effect which then contribute to the blood pressure. Table 4 shows the direct and indirect effects of risk factors associated with blood pressure for two groups. Obesity were associated with increase in blood pressure directly in women (ß = 0.15) and men (ß = 0.24). In men and women, the direct effect of SES on the blood pressure was negative (ß = -0.13 vs ß = -0.17, respectively). However, the indirect effect of SES was positive (ß = 0.04 in men vs. ß = 0.03 in women). The mediators for the indirect effect of SES on blood pressure were obesity, diabetes and Mets (Figs. 2 and 3). Diabetes was directly associated with an increase in blood pressure in women (ß = 0.03) but the association was not statistically significant in men. Mets were indirectly and inversely associated with blood pressure (ß = -0.04 vs ß = -0.04), in men and women, respectively. The mediators for the indirect effect of Mets on blood pressure were obesity, diabetes and lipid profile. The direct and indirect effects of Mets on mediators variables were negative. Having positive family history of hypertension especially in the first-degree relatives were associated with a higher BP in men and women. lipid profile had both direct (ß = 0.05 vs ß = 0.09) and indirect effect (ß = 0.08 vs ß = 0.04) in men and women, respectively (Table 4). The mediators for the indirect effect of lipid profile on blood pressure were obesity and diabetes. lipid profile had a strong positive and direct relationship with obesity in men and women (Table 4). Taking antihypertensive drugs had direct and inverse association with blood pressure (ß = -0.29 vs ß = -0.33) in men and women, respectively (Table 4).

**Table 4:** Direct and indirect effects derived from a SEM in people 35–65 from the RaNCD cohort study

Variables	**Male**			**Female**		
	Total effect **(95%CI)**	Direct effect **(95%CI)**	Indirect effect **(95%CI)**	Total effect **(95%CI)**	Direct effect **(95%CI)**	Indirect effect **(95%CI)**
Obesity –- > BP	0.24(0.21—0.27)	0.24(0.21—0.27)	––	0.15(0.12—0.18)	0.15(0.12—0.18)	––
Lipid profile–- > BP	0.13(0.10—0.16)	0.05(0.02—0.08)	0.08(0.06—0.09)	0.13( 0.10, 0.15)	0.09(0.07, 0.12)	0.04(0.02, 0.05)
SES –- > BP	-0.09(-0.13, -0.06)	-0.13(-0.17,—0.09)	0.04(0. 02, 0.05)	-0.14(-0.18, -0.06)	-0.17(-0.20, -0.13)	0.03( 0.02, 0.04)
Diabetes–- > BP	–-	–-	–-	0.03(0.02, 0.05)	0.03(0.02, 0.05)	–-
MET –- > BP	-0.04(-0.07, -0.01)	–-	-0.04(-0.07, -0.01)	-0.04(-0.07, -0.02)	––	-0.04(-0.07, -0.02)
HTNDRUG –- > BP	-0.29 (-0.32, -0.25)	-0.29 (-0.32, -0.25)	–-	-0.33 (-0.36, -0.30)	-0.33 (-0.36, -0.30)	–-
HTN family–- > BP	0.02(0.02, 0.05)	0.02(0.02, 0.05)	––	0.03(0.01, 0.06)	0.03(0.01, 0.06)	–-
MET –- > obesity	-0.11 (-0.15, -0.08)	-0.08 (-0.11, -0.05)	-0.03 (-0.04, -0.02)	-0.08 (-0.11, -0.05)	-0.07 (-0.10, -0.04)	-0.01 (-0.01, -0.03)
MET–- > lipid profile	-0.11 (-0.13, -0.07)	-0.11 (-0.13, -0.07)	–-	-0.07 (-0.09, -0.04)	-0.07 (-0.09, -0.04)	–-
MET–- > Diabetes	-0.06 (-0.09, -0.03)	-0.06 (-0.09 -0.03)	–-	-0.03(-0.09, -0.03)	-0.02 (-0.05, -0.03)	-0.01 (-0.01, -0.03)
Lipidprofile–- > obesity	0.32(0.30, 0.35)	0.32(0.30, 0.35)	–-	0.21(0.18, 0.24)	0.21(0.18, 0.24)	–-
SES–- > METs	-0.39 (-0.41, -0.36)	-0.39 (-0.41, -0.36)	––	-0.23 (-0.26, -0.19)	-0.23 (-0.26, -0.19)	–-
SES–- > Obesity	0.13 (0.09, 0.16)	0.08 (0.04, 0.12)	0.05 (0.03, 0.06)	0.10 (0.06, 0.13)	0.08 (0.04, 0.12)	0.02 (0.01, 0.02)
SES–- > Lipid profile	0.04 (0.03, 0.05)	––-	0.04 (0.03, 0.05)	0.01 (0.01, 0.02)	––-	0.01 (0.01, 0.02)
Lipid profile –- > Diabetes	–-	–-	–-	0.04 (0.01,0.07)	0.04 (0.01,0.07)	–-

*SBP* Systolic blood pressure, *DBP* Diastolic blood pressure, *WHR* Waist to hip ratio, *BMI* Body mass index, *SES* Socioeconomic status, *WC* Waist circumference, *LDL* Low-density lipoproteins, *TC* Total cholesterol, *HDL* High-density lipoprotein, *HTNfamily* Family history of hypertension, *HTNdrug* Antihypertensive drug

## Discussion

Hypertension is a major public health problem globally [29]. Although many studies have examined the effect of risk factors for hypertension separately, only a few limited studies have evaluated the direct and indirect effects of associated factors with hypertension by considering the role of the mediator variables. The present study was conducted to determine the direct and indirect effects of modifiable and non-modifiable risk factors of hypertension using SEM.

This study has demonstrated that individual with a higher level of SES had a direct negative, and indirect positive effect (through obesity, MET, diabetes) on BP with positive effects on increasing the risk of dyslipidemia and obesity in both sexes. In addition, SES had total negative effect on BP. Increased awareness, accessibility of medical treatment and opportunities to prevent and diagnosis of hypertension have been indicated as protective effect of higher SES on of BP. However, unfavorable living habits, unhealthy diet including the consumption of high-calorie foods (the average energy consumed by our whole population was 3865.56 kcal/d, which was higher than the recommended levels), obesity and physical inactivity have been indicated as indirect positive effect of higher SES on raised blood pressure. This finding is consistent whit previous research that found that individual with a higher level of SES had a direct and indirect effect on hypertension with structural equation model [14, 16]. Individuals with high level of SES should pay more attention to prevent the hypertension, since SES as a distal risk factor, indirectly influenced obesity, MET, Diabetes, dyslipidemia through this pathway.

Obesity was the most important risk factor that directly affected the of BP in our study in both sexes which is consistent with the results of previous studies [14, 30]. Overall, 27.6% of all participants of RaNCD cohort study had a normal BMI, and the majority were overweight or obese, especially among women. For waist-to-hip ratio, there was a big difference between men and women. From a total of 9705 people (82.4%) with an abnormal value, 60.4% and 39.6% were women and men, respectively [21]. Obesity is a major risk factor for hypertension, and the association between hypertension and obesity has been confirmed in the past two decades. The combination of obesity and higher BP leads to an increased risk of developing cardiovascular complications [31].

In the present study, dyslipidemia was directly associated with of BP in both sexes, and was also associated directly with obesity in both men and women with direct association with diabetes in women, resulting in total positive effect (ß = 0.13 in both sexes). Many clinical studies have shown that the dyslipidemia is a strong marker for predicting the risk of atherosclerosis and heart diseases [32, 33]. In most studies, old age, hypertension and obesity were significantly associated with an abnormal lipid profiles [34]. The presence of hyperlipidemia is known to be a prognostic risk factor in patients with hypertension [13, 35].

The association between DM and serum lipids has been much debated over the past decades [36, 37]. Type 2 DM (T2DM) is usually associated with abnormal levels of serum lipids. The interaction between impaired lipid metabolism and blood sugar plays an important role in the onset and progression of diabetes and related chronic complications [38, 39]. In the present study, dyslipidemia was directly associated with diabetes in women. Higher level of physical activity are associated with a decreased risk of CVD [40]. We found that higher levels of physical activity had negative indirect effect (without direct effect), through decrease of mediator variables (obesity, dyslipidemia, Diabetes), on BP which are consistent with other reports [16, 30].

### Strengths and weaknesses

This study has strengths and weaknesses. The most important strength of the present study was the sample size which was large enough to investigate the association between all the above mentioned variables with hypertension. Our study reveals advantages of SEM application for of BP compared with the traditional analysis methods. In fact, SEM includes causal modeling, analysis of covariance structures, latent variable and robust model. SEM reduces measurement errors by involvement of several overt variables for each latent variable instead of single-measurement particularly with variables that are measured by multiple indicators, e.g.(SES, obesity, serum lipids) [41]. However, our study is cross-sectional in nature, which challenges the causal relationship between the variables. As well as the nature of BP which more than 90% of its causes are unknown, it was impossible to draw a model with a large number of variables due to the limitations of working with the software and SEM.

Our study showed that although there are other factors associated with of BP, among the modifiable risk factors, obesity and antihypertensive drugs had the strongest direct effect on the outcome. For future works, we suggest investigation of relationship between different factors with incidence of hypertension which is more valuable than prevalence.

## Conclusion

The present study demonstrated that BP was related to SES, physical activity, mean serum lipids, obesity and diabetes directly and (or) indirectly.In accordance with other reports, the public health interventions to prevent and control hypertension, should focus on obesity, increasing physical activity and improving the life style specifically among those with higher SES.

## Data Availability

The RaNCD cohort is not an open-access database. However, we would encourage external investigators to consider applying to use the data for secondary analyses, to maximize the scientific output from the data. All the information on how to access the RaNCD public data archive, with a list of current proposals and papers under preparation, can be found on our website: www.persiancohort.com
